# Successful implementation of a longitudinal skill-based teaching curriculum for residents

**DOI:** 10.1186/s12909-021-02765-x

**Published:** 2021-06-15

**Authors:** Jane Rowat, Krista Johnson, Lisa Antes, Katherine White, Marcy Rosenbaum, Manish Suneja

**Affiliations:** 1 Department of Internal Medicine, University of Iowa Hospitals and Clinics, Iowa City, IA 52242 USA; 2 Department of Family Medicine, University of Iowa Hospitals and Clinics, Iowa City, IA USA

**Keywords:** Graduate medical education, Resident teaching skills, Longitudinal skills-based teaching curriculum, Teaching curriculum

## Abstract

**Background:**

Despite significant teaching responsibilities and national accreditation standards, most residents do not receive adequate instruction in teaching methods. Published reports of residents-as-teachers programs vary from brief one-time exposures to curricula delivered over several months. A majority of interventions described are one or two-day workshops with no clear follow-up or reinforcement of skills. A three-year longitudinal teaching skills curriculum was implemented with these goals: 1) deliver an experiential skill-based teaching curriculum allowing all residents to acquire, practice and implement specific skills; 2) provide spaced skills instruction promoting deliberate practice/reflection; and 3) help residents gain confidence in their teaching skills.

**Methods:**

One hundred percent of internal medicine residents (82/82) participated in the curriculum. Every 10 weeks residents attended a topic-specific experiential skills-based workshop. Each workshop followed the same pedagogy starting with debriefing/reflection on residents’ deliberate practice of the previously taught skill and introduction of a new skill followed by skill practice with feedback. Every year, participants completed: 1) assessment of overall confidence in each skill and 2) retrospective pre-post self-assessment. A post-curriculum survey was completed at the end of 3 years.

**Results:**

Residents reported improved confidence and self-assessed competence in their teaching skills after the first year of the curriculum which was sustained through the three-year curriculum. The curriculum was well received and valued by residents.

**Conclusions:**

A formal longitudinal, experiential skills-based teaching skills curriculum is feasible and can be delivered to all residents. For meaningful skill acquisition to occur, recurrent continuous skill-based practice with feedback and reflection is important.

**Supplementary Information:**

The online version contains supplementary material available at 10.1186/s12909-021-02765-x.

## Background

Residents spend significant time on teaching activities regardless of their specialty or future career plans [1, 2], often having primary responsibility for teaching medical students on inpatient wards. The Accreditation Council for Graduate Medical Education (ACGME) and the Liaison Committee on Medical Education (LCME) require residents receive training to develop teaching skills, but do not specify content or implementation of skill development [3, 4]. Despite significant teaching responsibilities and national accreditation standards, most residents do not receive adequate skill-based instruction in teaching methods [2, 5].

Some studies indicate that resident teaching programs can be effective in improving self-reported behavior and confidence to teach [6–8]. Published reports of residents-as-teachers (RAT) programs vary from brief one-time exposures to curricula delivered over several months [9, 10]. A majority of interventions described are one or two-day teaching skills workshops with no clear follow-up or reinforcement of these skills [9, 10]. However, short interventions often do not lead to lasting improvements in teaching ability, and decrements in teaching skills have been reported without periodic reinforcement [7, 11].

There is a paucity of literature describing longitudinal skill-based teaching curricula with recurring re-enforcement that spans the entirety of residency training and includes all residents in a program rather than just selected learners interested in teaching. To address this need we implemented a three-year longitudinal teaching skills curriculum with these goals: 1) deliver an experiential skill-based teaching curriculum allowing all residents to acquire, practice and implement specific skills for effective teaching; 2) provide recurring spaced teaching skills instruction promoting deliberate practice and reflection; and 3) help residents gain confidence in their teaching skills. Here we describe the design, implementation, evaluation and effectiveness of this curriculum.

## Methods

### Design and implementation

**Fig. 1:**
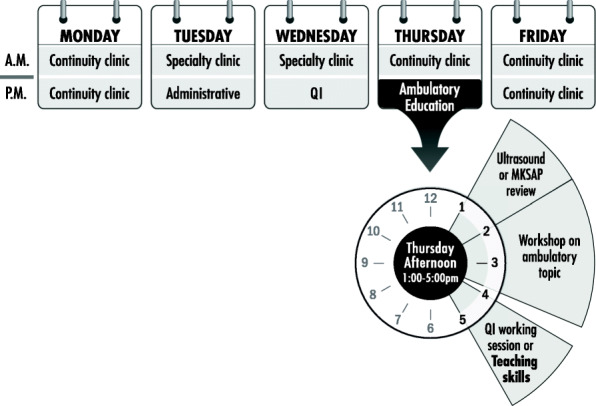
Sample “Y” week schedule

Prior to implementing this curriculum, the Department of Internal Medicine (IM) offered a “Teaching Resident Rotation” to third-year residents in which teaching experiences were concentrated into 1 month and only accommodated one-third of the cohort. In July 2015 the residency program adopted a 4 + 1 (X + Y) schedule which allowed implementation of a longitudinal 3-year teaching skills curriculum for all residents (Fig. 1) [12].

The curriculum was designed and implemented using a six-step approach to curriculum development [13]. In the planning phase the Resident Teaching Skills Committee comprised of educators and residents from the Department of IM as well as medical education experts from the Office of Consultation and Research in Medical Education (OCRME) conducted a review of RAT literature [7, 9, 10, 14]. The committee created a list of teaching topics relevant to residents’ teaching responsibilities and opportunities. Each member independently ranked topics in order of importance (Supplemental Table 1). The most highly ranked topics were discussed, and fifteen teaching skills were chosen to be delivered over 3 years. Teaching skills sessions with associated learning objectives are listed in Table 1. Every 10 weeks residents participated in a program required topic-specific experiential skills-based workshop (60 min) which utilized various educational strategies including video-taped scenarios, small-group discussions and role-play. These strategies helped us to incorporate deliberate practice and reflection in the workshops. Facilitators of the teaching skills sessions were experts in the content area specific to the session. If additional facilitators were required for skill practice, they were trained by the session experts.

**Table 1:** Teaching skills sessions with associated learning objectives and activities

Year One Curriculum (2015–2016)	
**Teaching Skills Topic**	**Learning Objectives: as a result of this session, residents will be able to:**
Introduction to Resident Teaching Skills Curriculum, Learning Climate and Effective Teachers	• Identify characteristics of exemplary clinical teachers
	• Identify strategies to promote an effective learning climate
	• Demonstrate teaching behaviors that promote an effective learning climate
Motivating the Learner	• Examine contributing factors that affect a learner’s performance
	• Explain one relevant motivational theory that covers extrinsic and intrinsic motivators
	• Discuss the importance and impact of a good orientation
	• Identify components of an orientation
One-Minute Preceptor	• Describe the five elements of the One-Minute Preceptor model for clinical teaching
	• Successfully apply the model to a simulated learner presenting a patient
	• Use the model to develop an assessment of the learner’s current level of knowledge/skill and what the learner needs to know
Effective Feedback	• Define feedback and give rationale for providing feedback to learners
	• Recognize barriers to giving feedback
	• Identify characteristics of effective feedback
	• Demonstrate effective feedback via observation and practice
Interactive Teaching/Use of Technology	• List goals of effective lecturing/presentations
	• Describe components of effective lecturing/presentation
	• Apply specific techniques for making lectures more interactive
**Year Two Curriculum (2016–2017)**	
**Teaching Skills Topic**	**Learning Objectives: as a result of this session, residents will be able to:**
Introduction to Teaching Skills, Learning Climate and Effective Teachers	• Identify ways to utilize Carver College of Medicine and clerkship learning objectives for medical students in bedside and small group teaching
	• Formulate an orientation checklist for outlining logistics and student expectations on the service
	• Recognize leadership and teaching behaviors that create an environment of harassment and learner mistreatment.
Senior Resident Curriculum: Anatomy of an Effective Presentation	• Recognize steps involved in developing an effective presentation
	• Understand importance of creating a timeline
	• Describe structure of an effective presentation
	• Identify tips and tools for an effective presentation
	• Design slides using presentation principles
Intern Teaching Primer: Introduction to Teaching Skills	• Appreciate impact of resident teachers on learners
	• Identify expectations for resident teachers
	• Recognize challenges in clinical teaching
	• Demonstrate teaching skills: orientation and feedback
Bedside Teaching Skills	• Recognize advantages of bedside teaching
	• Discuss barriers to bedside teaching
	• Identify strategies for effectively teaching in the patient’s presence
Teaching Clinical Reasoning	• Recognize level of learner you are working with on rotations
	• Develop questions appropriate to level of learner
	• Assess learner’s diagnostic reasoning ability
	• Practice giving feedback to learner and developing an educational plan
Post-Curriculum Feedback and Survey	• Discuss personal growth as a teacher
	• Share teaching experiences and application of teaching skills learned over the course of the year
**Year Three Curriculum (2017–2018)**	
**Teaching Skills Topic**	**Learning Objectives: as a result of this session, residents will be able to:**
Introduction to Teaching Skills, Learning Climate and Effective Teachers	• Identify ways to utilize Carver College of Medicine, subinternship and clerkship learning objectives for medical students in bedside and small group teaching.
	• Assist learners in setting learning goals for the clerkship.
	• Recognize leadership and teaching behaviors that create an environment of harassment and leaner mistreatment.
	• Identify people in the department who are resources if they need to report learner mistreatment
Senior Resident Curriculum: Engaging Presentations	• Recognize steps involved in developing an effective presentation
	• Understand importance of creating a timeline
	• Describe structure of an effective presentation
	• Identify tips and tools for an effective presentation
	• Integrate tips and tools into personal presentation
Intern Teaching Primer: Introduction to Teaching Skills	• Appreciate the impact of resident teachers on learners
	• Identify expectations for resident teachers
	• Recognize challenges in clinical teaching
	• Demonstrate teaching skills- orientation and feedback
Teaching and Assessing Oral Presentations	• Review basic guidelines of oral presentations, including timing, delivery and emphasis
	• Highlight the oral presentation as a form of medical communication
	• Differentiate features of oral vs written presentations
	• Demonstrate effective acquisition and delivery tips
	• Demonstrate how to assess representative oral presentations using a checklist
Teaching and Assessing Clinical Notes	• Learn to critically evaluate a history and physical written by a medical student
	• Learn to critically evaluate a progress note written by a medical student
	• Practice giving feedback to a medical student on their written notes
Post-curriculum Feedback and Surveys	• Differentiate between feedback and evaluation
	• Discuss the cycle of feedback and evaluation
	• Describe G.R.A.D.E. strategy for evaluation
	• Review the Carver College of Medicine clinical student evaluation form

**Fig. 2:**
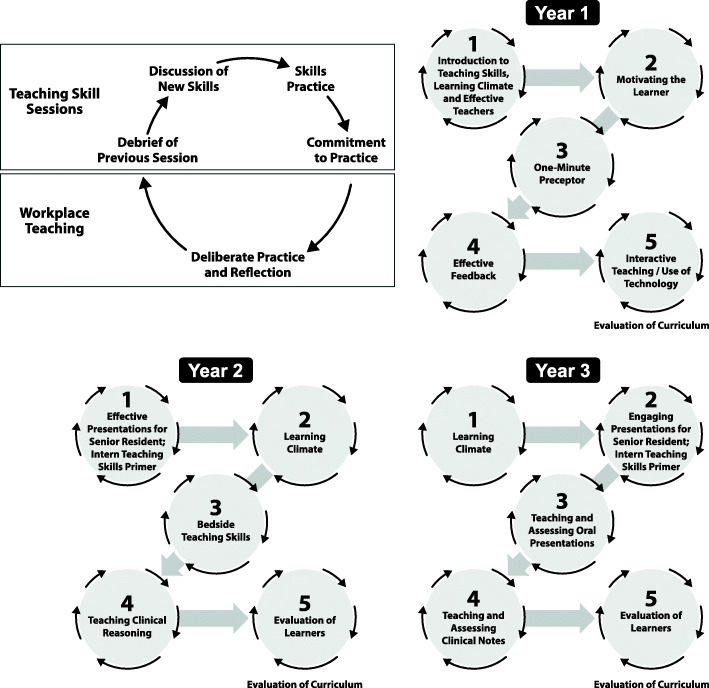
Pedagogy and topics utilized for teaching skills sessions

These instructional strategies helped us to incorporate skill practice, deliberate practice and reflection. Each workshop followed the same pedagogy (Fig. 2) starting with debriefing/ reflection on residents’ deliberate practice of the previously taught skill and introduction of a new skill followed by practice with feedback from clinician educators and peers. To promote deliberate practice, before leaving the workshop every resident committed to a personal action plan aimed at furthering skill development related to that session topic. Pocket cards summarizing key take-home points were distributed at the end of each session (Supplemental Table 2A-H). Residents were expected to attempt to implement their personal action plan and these experiences were debriefed allowing for individual/group reflection to deepen learning and efficacy in using these skills. Pocket cards with workshop learning objectives were distributed electronically to all departmental faculty allowing them to reinforce and provide workplace-based feedback on the teaching skill residents were practicing. (Supplemental Table 3).

### Study participants

All IM and Medicine-Psychiatry residents (82/82; 100%) participated in the curriculum.

Overall data was collected for 3 years; PGY1, PGY2 and PGY3 cohort data was collected for 3, 2 and 1 year, respectively. Participants completed a curricular satisfaction at the end of 3 years (Supplemental Table 7).

### Data collection and measurement instruments

Several measures were used to assess characteristics of participants and perceived impact of the curriculum on learners’ confidence and skills. Information on baseline experience and teaching interest (Supplemental Table 4) was collected from all residents at the beginning of Year One. At the end of each year (for 3 years), participants completed: 1) assessment of overall confidence in using each teaching skill (Supplemental Table 5); and 2) retrospective pre-post self-assessment comparing their perceived competence with each teaching skill at the end of each year. (Supplemental Table 6). Response to the Association of American Medical Colleges (AAMC) Graduation Questionnaire (GQ) survey asking medical students to evaluate IM resident teaching effectiveness during their clerkships was reviewed prior to and after implementation of the teaching skills curriculum to further assess impact of the curriculum.

### Data analysis

Confidence and self-assessed competence in performing each teaching skill was assessed using a 5-point scale (“4” or “5” considered confident and self-assessed competence). Wilcoxon signed-rank test was used to test change in response at each post-assessment compared to baseline and compared to prior year post-assessment. Jonckheere-Terpstra test for ordered differences was used to assess the differences in confidence scores among program years. Statistical analyses were performed using SAS version 9.4.

This project was deemed non-human-subjects research by the Institutional Review Board of the University of Iowa.

## Results

### Baseline data

The baseline survey was completed by 92% (75/82) of participants. Eighty-nine percent (67/75) of residents indicated interest in teaching, 77% (58/75) anticipated teaching will be part of their career and 25% (19/75) reported previous participation in a formal teaching course.

### Confidence data

Confidence scores after 1 year of curriculum indicated majority of residents felt confident in their teaching skills (Supplemental Table 8A). Confidence scores (creating a positive learning environment, showing respect for learners and using wait time when questioning learners) showed significant improvement after 2 years of the curriculum (Supplemental Table 8B). There were significant differences noted in the confidence skills between PGY1s and PGY 2/3 s in four categories (dealing with challenging learners, facilitating a small group, providing feedback consistently and identifying important skills for teachers). The only significant confidence measure difference between PGY2/3 s after 2 years in the curriculum was facilitating a small group session. No degradation was seen in third-year residents’ self-reported confidence in teaching skills.

### Pre-Post self-assessment data

Data reported by PGY1s after participating in the curriculum for 1 year showed significant improvement in all self-assessed skills (Table 2). PGY2s reported significant improvement in all but three categories (engaging in discussion about medical issues, feeling comfortable stating “I am not sure” and showing respect for learners). PGY3s reported significant improvement in all categories except two (feeling comfortable stating “I am not sure” and showing respect for learners). Self-assessment of teaching skills after participating in the curriculum for 2 years continued to show significant improvement in all but one of the teaching skills in the PGY 2/3 s (showing respect for learners) (Table 3).

**Table 2:** Self-assessment: change score (pre/post) after first year of participation in curriculum by program year

Question	Program Year	n	(−) Decrease		(0) No change		(+) 1		(+) 2		(+) > 2		Ho: post/pre = 0 ***p***-value	***p***-value*
			Count	%	Count	%	Count	%	Count	%	Count	%		
Actively listen when student presents information	1	58	3	*5.2*	31	*53.4*	20	*34.5*	4	*6.9*	0	*0.0*	< 0.0001	0.817
	2	20	0	*0.0*	14	*70.0*	5	*25.0*	1	*5.0*	0	*0.0*	0.031	
	3	15	1	*6.7*	7	*46.7*	7	*46.7*	0	*0.0*	0	*0.0*	0.070	
Ask for feedback on my teaching skills, abilities	1	58	1	*1.7*	21	*36.2*	29	*50.0*	6	*10.3*	1	*1.7*	< 0.0001	0.288
	2	19	0	*0.0*	6	*31.6*	7	*36.8*	6	*31.6*	0	*0.0*	0.0002	
	3	15	0	*0.0*	5	*33.3*	7	*46.7*	3	*20.0*	0	*0.0*	0.002	
Ask questions that encourage learner to think about medical issue	1	58	1	*1.7*	13	*22.4*	34	*58.6*	9	*15.5*	1	*1.7*	< 0.0001	0.284
	2	20	1	*5.0*	6	*30.0*	12	*60.0*	1	*5.0*	0	*0.0*	0.010	
	3	15	1	*6.7*	4	*26.7*	7	*46.7*	3	*20.0*	0	*0.0*	0.009	
Choose appropriate methods for learning material	1	58	0	*0.0*	26	*44.8*	29	*50.0*	2	*3.4*	1	*1.7*	< 0.0001	0.406
	2	20	0	*0.0*	8	*40.0*	9	*45.0*	3	*15.0*	0	*0.0*	0.0005	
	3	17	0	*0.0*	6	*35.3*	10	*58.8*	1	*5.9*	0	*0.0*	0.001	
Clearly communicate information about student performance during rotation	1	58	0	*0.0*	19	*32.8*	26	*44.8*	12	*20.7*	1	*1.7*	< 0.0001	0.518
	2	20	0	*0.0*	5	*25.0*	10	*50.0*	5	*25.0*	0	*0.0*	0.0001	
	3	15	0	*0.0*	4	*26.7*	7	*46.7*	3	*20.0*	1	*6.7*	0.001	
Coach through new procedures instead of doing them myself	1	57	0	*0.0*	38	*66.7*	13	*22.8*	5	*8.8*	1	*1.8*	< 0.0001	0.054
	2	20	0	*0.0*	9	*45.0*	10	*50.0*	1	*5.0*	0	*0.0*	0.001	
	3	15	0	*0.0*	7	*46.7*	4	*26.7*	3	*20.0*	1	*6.7*	0.008	
Convey expectations for learning, performance, behavior	1	58	0	*0.0*	14	*24.1*	35	*60.3*	9	*15.5*	0	*0.0*	< 0.0001	0.764
	2	20	0	*0.0*	4	*20.0*	11	*55.0*	5	*25.0*	0	*0.0*	< 0.0001	
	3	17	0	*0.0*	6	*35.3*	9	*52.9*	2	*11.8*	0	*0.0*	0.001	
Create positive, supportive learning environment	1	58	0	*0.0*	31	*53.4*	21	*36.2*	6	*10.3*	0	*0.0*	< 0.0001	0.574
	2	20	0	*0.0*	14	*70.0*	5	*25.0*	1	*5.0*	0	*0.0*	0.031	
	3	17	1	*5.9*	7	*41.2*	8	*47.1*	1	*5.9*	0	*0.0*	0.076	
Demonstrate interest in teaching, allot time for it	1	58	1	*1.7*	19	*32.8*	28	*48.3*	10	*17.2*	0	*0.0*	< 0.0001	0.449
	2	19	0	*0.0*	9	*47.4*	9	*47.4*	1	*5.3*	0	*0.0*	0.002	
	3	17	0	*0.0*	6	*35.3*	9	*52.9*	2	*11.8*	0	*0.0*	0.001	
Discuss learner’s goals during the rotation	1	58	0	*0.0*	19	*32.8*	31	*53.4*	7	*12.1*	1	*1.7*	< 0.0001	0.682
	2	20	0	*0.0*	3	*15.0*	13	*65.0*	3	*15.0*	1	*5.0*	< 0.0001	
	3	17	0	*0.0*	7	*41.2*	7	*41.2*	3	*17.6*	0	*0.0*	0.002	
Engage in discussions about medical issues	1	58	1	*1.7*	22	*37.9*	31	*53.4*	4	*6.9*	0	*0.0*	< 0.0001	0.571
	2	20	1	*5.0*	12	*60.0*	6	*30.0*	1	*5.0*	0	*0.0*	0.188	
	3	15	0	*0.0*	6	*40.0*	6	*40.0*	3	*20.0*	0	*0.0*	0.004	
Feel comfortable stating “I am not sure” when I do not know answer	1	58	0	*0.0*	41	*70.7*	11	*19.0*	6	*10.3*	0	*0.0*	< 0.0001	0.035
	2	20	1	*5.0*	17	*85.0*	2	*10.0*	0	*0.0*	0	*0.0*	1.00	
	3	15	0	*0.0*	13	*86.7*	2	*13.3*	0	*0.0*	0	*0.0*	0.500	
Give frequent, constructive feedback	1	58	1	*1.7*	15	*25.9*	31	*53.4*	10	*17.2*	1	*1.7*	< 0.0001	0.199
	2	20	0	*0.0*	3	*15.0*	11	*55.0*	6	*30.0*	0	*0.0*	< 0.0001	
	3	15	0	*0.0*	4	*26.7*	6	*40.0*	4	*26.7*	1	*6.7*	0.001	
Provide opportunity to observe, participate in clinically relevant procedures	1	58	0	*0.0*	26	*44.8*	27	*46.6*	5	*8.6*	0	*0.0*	< 0.0001	0.475
	2	20	0	*0.0*	11	*55.0*	5	*25.0*	4	*20.0*	0	*0.0*	0.004	
	3	15	0	*0.0*	8	*53.3*	7	*46.7*	0	*0.0*	0	*0.0*	0.016	
Show support, respect for learners	1	58	0	*0.0*	44	*75.9*	13	*22.4*	1	*1.7*	0	*0.0*	< 0.0001	0.900
	2	20	1	*5.0*	12	*60.0*	6	*30.0*	1	*5.0*	0	*0.0*	0.188	
	3	17	0	*0.0*	14	*82.4*	3	*17.6*	0	*0.0*	0	*0.0*	0.250	

*p-value from Wilcoxon rank-sum exact test

**Table 3:** Self-assessment: change score (pre/post) after second year participation in curriculum by program year

Statement	Program Year	n	(−) Decrease		(0) No change		(+) 1		(+) 2		(+) > 2		Ho: post/pre = 0 ***p***-value	***p***-value*
			Count	%	Count	%	Count	%	Count	%	Count	%		
Actively listen when student presents information	2	31	2	*6.5*	11	*35.5*	16	*51.6*	2	*6.5*	0	*0.0*	0.0003	0.704
	3	16	0	*0.0*	9	*56.3*	5	*31.3*	2	*12.5*	0	*0.0*	0.016	
Ask for feedback on my teaching skills, abilities	2	31	1	*3.2*	12	*38.7*	15	*48.4*	2	*6.5*	1	*3.2*	0.0001	0.374
	3	16	0	*0.0*	5	*31.3*	8	*50.0*	3	*18.8*	0	*0.0*	0.001	
Ask questions that encourage learner to think about medical issue	2	31	0	*0.0*	12	*38.7*	16	*51.6*	2	*6.5*	1	*3.2*	< 0.0001	0.656
	3	16	0	*0.0*	9	*56.3*	3	*18.8*	4	*25.0*	0	*0.0*	0.016	
Choose appropriate methods for learning material	2	31	0	*0.0*	13	*41.9*	16	*51.6*	2	*6.5*	0	*0.0*	< 0.0001	0.667
	3	16	0	*0.0*	5	*31.3*	11	*68.8*	0	*0.0*	0	*0.0*	0.001	
Clearly communicate information about student performance during rotation	2	31	1	*3.2*	11	*35.5*	14	*45.2*	3	*9.7*	2	*6.5*	< 0.0001	0.300
	3	16	0	*0.0*	3	*18.8*	10	*62.5*	3	*18.8*	0	*0.0*	0.0002	
Coach through new procedures instead of doing them myself	2	31	0	*0.0*	14	*45.2*	16	*51.6*	1	*3.2*	0	*0.0*	< 0.0001	0.782
	3	16	0	*0.0*	9	*56.3*	5	*31.3*	2	*12.5*	0	*0.0*	0.016	
Convey expectations for learning, performance, behavior	2	31	0	*0.0*	5	*16.1*	22	*71.0*	4	*12.9*	0	*0.0*	< 0.0001	0.822
	3	16	0	*0.0*	2	*12.5*	12	*75.0*	1	*6.3*	1	*6.3*	0.0001	
Create positive, supportive learning environment	2	31	0	*0.0*	18	*58.1*	11	*35.5*	2	*6.5*	0	*0.0*	0.0002	0.996
	3	16	0	*0.0*	9	*56.3*	6	*37.5*	1	*6.3*	0	*0.0*	0.016	
Demonstrate interest in teaching, allot time for it	2	31	0	*0.0*	13	*41.9*	15	*48.4*	3	*9.7*	0	*0.0*	< 0.0001	0.917
	3	16	0	*0.0*	8	*50.0*	4	*25.0*	4	*25.0*	0	*0.0*	0.008	
Discuss learner’s goals during the rotation	2	31	0	*0.0*	13	*41.9*	10	*32.3*	8	*25.8*	0	*0.0*	< 0.0001	0.840
	3	16	0	*0.0*	4	*25.0*	12	*75.0*	0	*0.0*	0	*0.0*	0.0005	
Engage in discussions about medical issues	2	31	0	*0.0*	9	*29.0*	18	*58.1*	3	*9.7*	1	*3.2*	< 0.0001	0.923
	3	16	0	*0.0*	4	*25.0*	10	*62.5*	2	*12.5*	0	*0.0*	0.0005	
Feel comfortable stating “I am not sure” when I do not know answer	2	31	0	*0.0*	19	*61.3*	10	*32.3*	2	*6.5*	0	*0.0*	0.0005	0.780
	3	16	0	*0.0*	9	*56.3*	6	*37.5*	1	*6.3*	0	*0.0*	0.016	
Give frequent, constructive feedback	2	31	0	*0.0*	9	*29.0*	18	*58.1*	4	*12.9*	0	*0.0*	< 0.0001	0.883
	3	16	0	*0.0*	6	*37.5*	7	*43.8*	3	*18.8*	0	*0.0*	0.002	
Provide opportunity to observe, participate in clinically relevant procedures	2	31	0	*0.0*	15	*48.4*	10	*32.3*	6	*19.4*	0	*0.0*	< 0.0001	0.301
	3	16	0	*0.0*	10	*62.5*	5	*31.3*	1	*6.3*	0	*0.0*	0.031	
Show support, respect for learners	2	31	1	3.2	23	74.2	7	22.6	0	0	0	0	0.070	0.916
	3	16	0	0	13	81.3	3	18.8	0	0	0	0	0.250	

*p-value from Wilcoxon rank-sum exact test

### AAMC graduation questionnaire data

Following the implementation of the curriculum IM residents’ ratings by medical students on the question *“Residents provide effective teaching during the clerkship”* significantly exceeded all medical schools (97.6% Carver College of Medicine students agreed/strongly agreed vs 92.9% for all medical schools) as compared to prior to implementation ratings (93.5% Carver College of Medicine students agreed/ strongly agreed vs 93.3% for all medical schools (Supplemental Table 9).

### Curricular evaluation data

Curriculum evaluation was completed by 67% (55/82) participants. Residents rated the curriculum highly and 82% (45/55) thought it provided them with longitudinal comprehensive teaching skills and 75% (41/55) reported it provided a comprehensive program for their development as a teacher. Only 49% (27/55) reported getting feedback from faculty on teaching skills being taught during the curriculum (Table 4).

**Table 4:** Teaching skills curriculum evaluation

Question	Strongly disagree (1)	2	3	4	Strongly agree (5)
The amount of work I was expected to complete was reasonable	0	2	12	24	17
	0	3.64	21.82	43.64	30.91
I found the practice/role play during the sessions to be helpful in learning specific teaching skills being emphasized	0	2	16	23	14
	0	3.64	29.09	41.82	25.45
The pocket cards summarized the teaching skills being highlighted	0	2	18	22	13
	0	3.64	32.73	40	23.64
I refer to the cards in my teaching	0	3	11	22	19
	0	5.45	20	40	34.55
The topics chosen were helpful in my development as a teacher	0	1	19	30	5
	0	1.82	34.55	54.55	9.09
The sessions provided me with a longitudinal comprehensive teaching skills curriculum	0	1	9	36	9
	0	1.82	16.36	65.45	16.36
The “Y” week teaching skills curriculum provides a comprehensive program for my development as a teacher	0	2	12	28	13
	0	3.64	21.82	50.91	23.64
I have seen the teaching skills role modeled by faculty	0	1	8	27	19
	0	1.82	14.55	49.09	34.55
Faculty have provided feedback on the teaching skills I was introduced to during the Resident Teaching Skill curriculum	2	7	19	24	3
	3.64	12.73	34.55	43.64	5.45

*n* = 55; distribution of responses, count/percent of n

## Discussion

Based on accreditation standards, residents must be adequately prepared to perform their teaching responsibilities [3, 4]. Irrespective of the external standards, residency programs have a vested interest in developing residents into effective teachers. In a national survey of residency program directors, 55% reported their programs offered residents formal teaching skills instruction [5]. While most of our residents expressed interest in teaching, the majority lacked previous experience in a formal teaching curriculum.

We successfully implemented a longitudinal three-year skill-based curriculum which incorporated skill practice, deliberate practice and reflection. Continuous skills-based practice and feedback is vital for acquisition and maintenance of any skill [15]. Having a dedicated block of time enabled this longitudinal teaching skills curriculum to be delivered to all IM residents on a scheduled, recurring basis. This allowed residents to engage in formal teaching skills practice and reflection over regular predictable intervals which likely enhanced their skill acquisition and confidence. Wamsley et al. and Edwards et al. have argued for the importance of reinforcing teaching principles to prevent degradation of teaching skills over time [7, 11]. This longitudinal structure for curricular implementation is also supported by a study which demonstrated that ‘spaced education’ (educational encounters spread out and repeated over time) improved learner retention of skills/curricular material [16]. While it is not possible to know whether interest and satisfaction would remain high without the teaching skill curriculum, it is likely that the curriculum may contribute to sustaining enthusiasm for teaching by providing on-going mentored support, practice and discussion. Skill improvement in all cohorts suggests that this longitudinal curriculum helped minimize skill degradation.

Residents reported improved confidence and self-assessed competence in their teaching skills after the first year of the curriculum and this was sustained through the three-years. The curriculum was well received and valued by our residents. Comparison of pre and post intervention responses to the AAMC GQ for effective clerkship teaching by IM residents also showed improvement in the residents’ ratings as effective teachers.

There are several possible limitations to our findings. It was conducted at one program in a single university setting which limits its generalizability. Teaching skill confidence and competence were assessed by self-report. However, the results were strengthened by the fact they compared the same individual’s progression in the curriculum over 3 years. The confidence and self-assessment surveys are self-administered instruments, making them subject to social desirability biases. The retrospective pre-post format was chosen for the self-assessment of skill competence to minimize response shift bias. This bias can underestimate program effectiveness in traditional pre-post surveys because participants may overestimate their knowledge prior to training [17, 18]. This study involved different cohorts over a three-year period who may have different characteristics (previous teaching skills training as well as teaching experiences), though the results presented followed the same individuals’ progression through the curriculum. In addition, it was an educational intervention study where the cohort with more time on task would generally be expected to have better outcomes. Therefore, a more accurate picture of teaching skill ability would require an objective measurement of teaching skills as well as work-place based assessment. Using Objective Structured Teaching Exercise (OSTE) performance data for the skills taught in the curriculum would be an objective way to assess the utility of our intervention.

Some valuable lessons were learned during the implementation of this curriculum. First-year residents are now provided a separate session to orient them to the curriculum. To ensure that all residents receive a similar experience, a cohort of motivated faculty are required to deliver the same content for five consecutive weeks. For meaningful skill acquisition continuous skill-based practice and feedback is important. Only half of the residents who responded to the survey reported getting feedback from faculty regarding their teaching skills in the workplace. This suggests more deliberate faculty development is needed rather than simply providing them with pocket cards and learning objectives electronically as done in our study. To more objectively study resident teaching skill acquisition and retention, we are in the process of designing and implementing annual OSTEs and incorporating workplace-based direct observation with feedback. Other tools which have been studied to successfully incorporate faculty feedback based on workplace-based observation include mini- CEX, clinical encounter cards, multi-source feedback and direct observation of teaching skills [19, 20].

## Conclusions

A formal longitudinal, experiential skills-based teaching skills curriculum is feasible and can be delivered to all residents. While IM residents expressed great interest in teaching, most had not participated in a formal teaching skills curriculum. Self-reported assessment data indicated improvement in resident confidence and teaching skills. Implementation of the curriculum is time-intensive and requires dedicated faculty. For meaningful skill acquisition to occur, recurrent continuous skill-based practice with feedback and reflection is important.

## Supplementary Information

**Additional file 1: Supplemental Table 1.** Teaching skills topic inventory.

**Additional file 2: Supplemental Table 2.** Workshop pocket cards.

**Additional file 3: Supplemental Table 3.** Email distribution of teachings skills information to faculty.

**Additional file 4: Supplemental Table 4.** Resident baseline experience and teaching interest.

**Additional file 5: Supplemental Table 5.** Teaching skills confidence survey.

**Additional file 6: Supplemental Table 6.** Teaching skills self-assessment.

**Additional file 7: Supplemental Table 7.** Teaching skills curriculum evaluation.

**Additional file 8: Supplemental Table 8A.** Confidence score after first year of participation in curriculum by program year. **Supplemental Table 8B.** Confidence score after second year of participation in curriculum by program year.

**Additional file 9: Supplemental Table 9.** AAMC graduation questionnaire: “Residents provide effective teaching during clerkship.”

## Data Availability

The datasets generated during and/or analyzed are available from the corresponding authors on reasonable request.

## Abbreviations

ACGME: Accreditation Council for Graduate Medical Education
LCME: Liaison Committee on Medical Education
RAT: Residents-as-Teacher
IM: Internal Medicine
OCRME: Office of Consultation and Research in Medical Education
PGY1: Postgraduate Year One
PGY2: Postgraduate Year Two
PGY3: Postgraduate Year Three
AAMC: Association of American Medical Colleges
GQ: Graduation Questionnaire
OSTE: Objective Structured Teaching Exercise
