# Supplementary material for: Successful implementation of a longitudinal skill-based teaching curriculum for residents
Source: BMC Med Educ. 2021 Jun 15;21:346. doi: 10.1186/s12909-021-02765-x (PMC8207581; doi:10.1186/s12909-021-02765-x)
Supplement: Supplementary file 1 — Additional file 1: Supplemental Table 1. Teaching skills topic inventory. [file 12909_2021_2765_MOESM1_ESM.docx]

**Supplemental Table 1.** Teaching skills topic inventory.

| Adult learning theory | Interactive teaching (techniques, stories/anecdotes, surveying student knowledge |
| --- | --- |
| Ability to identify gaps in knowledge and provide guidance for self-directed learning | Large group/community teaching |
| Assessing learners’ needs | Learning climate/learning environment |
| Brief “chalk talks” on topic | Medical education databases to search for teaching methods and curriculum |
| Characteristics of effective teachers | One-minute preceptor (microskills) |
| Clear goals and learning objectives for various teaching modalities | One-on-one teaching |
| Colleague education (faculty, residents, students, other healthcare professionals) | Patient education/communication at appropriate level |
| Creating a teaching philosophy | Physical exam skills (Stanford 25; DeGowin Physical Diagnosis; observing and critiquing physical exams; mini-CEX) |
| Creating a teaching portfolio | Promotion of understanding and retention |
| Develop teaching module using basic elements of curriculum design | Role modeling |
| Effective explanations | Scholarship/projects |
| Effective presentation skills (interactive lecturing/presentation skills) | Simulation (time-out technique) |
| Engaging all learners | Small group teaching |
| Evaluate article from the medical education literature | Stanford Seven |
| Evaluation of learners | Teaching in presence of the patient (inpatient and outpatient) |
| Feedback (understand and use various types of feedback) | Teaching in a variety of environments: rounding, bedside, lecture style |
| History taking skills | Team management skills-leading a team |
| Incorporating other skills into teaching including evidence-based medicine, basic science | Technology (use of PowerPoint, projected visuals, etc.). |
|  | Time management skills |
