# Supplementary material for: Successful implementation of a longitudinal skill-based teaching curriculum for residents
Source: BMC Med Educ. 2021 Jun 15;21:346. doi: 10.1186/s12909-021-02765-x (PMC8207581; doi:10.1186/s12909-021-02765-x)
Supplement: Supplementary file 2 — Additional file 2: Supplemental Table 2. Workshop pocket cards. [file 12909_2021_2765_MOESM2_ESM.pdf]

Supplemental Table 2. Workshop pocket cards.

Learning Climate

Internal Medicine Education Center — The University of Iowa

Learning Climate

The tone or atmosphere of the clinical teaching setting, including whether it is stimulating and whether learners can comfortably identify and address their limitations.

TIMING of teacher behaviors (initial, ongoing)

KEY COMPONENTS OF TEACHING BEHAVIORS

STIMULATION
- Show enthusiasm for topic and learners
- Show interest through body language
- Use animated voice
- Provide conducive physical environment

LEARNER INVOLVEMENT
- Look at learners
- Listen to learners
- Encourage learners to participate
- Avoid monopolizing discussion

RESPECT/COMFORT
- Use learner's name
- Acknowledge learners' problems/situation
- Avoid ridicule, intimidation, interruption

ADMISSIONS OF LIMITATIONS
- Acknowledge learner limitations
- Invite learners to bring up problems
- Admit own errors or limitations
- Avoid being dogmatic

Copyright 1989, Stanford University Faculty Development Program, Stanford University School of Medicine

Internal Medicine Education Center — The University of Iowa

Learning Climate Action Plan

List 1-2 skills related to learning climate that you would like to improve upon and try when working with learners before the next teaching session.

1.

2.

One-Minute Preceptor

Internal Medicine Education Center — The University of Iowa

The One-Minute Preceptor
Clinical Teaching Method

Diagnosis of the Learner

1. **Get a commitment**
   Ask learner to articulate own hypotheses or plan: "What do you think is going on with the patient?" "What other investigations should be ordered?"

2. **Probe for supporting evidence**
   Evaluate learner's knowledge or reasoning: "What on the physical exam makes you think this is CHF?"

Teaching

3. **Reinforce what was well done**
   Positive feedback: "You did a good job of considering multiple diagnoses and prioritizing them."

4. **Correct errors**
   Constructive feedback with instructions for improvement: "Remember to use the bell of your stethoscope when listening for bruits."

5. **Teach general rules (take-home points)**
   Particularly directed at areas of weakness for learner: "Remember to consider empyema in a patient with non-resolving pleural effusion and fever."

---

1. Gallagher P et al. Clin Teach 2012;9(6):358-62.
2. Neher JO et al. J Am Board Fam Pract 1992;5(4):419-24.

Internal Medicine Education Center — The University of Iowa

The One-Minute Preceptor

Diagnosis of the Learner

1. **Get a commitment**
   Ask learner to articulate own hypotheses or plan

2. **Probe for supporting evidence**
   Evaluate learner's knowledge or reasoning

Teaching

3. **Reinforce what was well done**
   Positive feedback

4. **Correct errors**
   Constructive feedback with instructions for improvement

5. **Teach general rules (take-home points)**
   Particularly directed at areas of weakness for learner

What would you like to try?

Feedback

Internal Medicine Education Center

THE UNIVERSITY OF IOWA

Providing Effective Feedback
Setting the Stage

- **Expected**
  I will be giving you feedback following this encounter. Shall we spend a few minutes discussing how the clinic went after your last patient?
- **Supportive learning climate**
  It's my job to help you improve.
- **Label as feedback**
  How would you like me to focus my feedback?
- **Self-assessment**
  What were you trying to accomplish during this encounter? Did it go as planned?

STOP

S: SPECIFIC
Your social history of the patient showed understanding of the pathophysiology and allowed us to focus more on biliary tract disease and less on alcoholism.

T: TIMELY
I'll give you feedback after each patient today. Let's plan to sit down at the end of the week.

O: OBJECTIVE, based on observed behaviors
I noticed you interrupted the patient a few seconds afer asking what brought her to the hospital. You lost eye contact with the patient when you were discussing the lab results.

P: PLAN for improvement
If we were going to do it again, what would you do the same and do differently? What actions do you want to take to improve in this area? How would you feel doing it again?

Gigante J, Dell M, Sharkey A. Getting beyond "good job": how to give effective feedback. Pediatrics 2011;127:205-7.

Internal Medicine Education Center

THE UNIVERSITY OF IOWA

Feedback Action Plan

List 1-2 skills related to feedback that you would like to improve upon and try when working with learners before the next teaching session.

1.

2.

Interactive Lecturing

Internal Medicine Education Center

The University of Iowa

Interactive Lecturing

**Attract Attention**	• Stories, anecdotes, cases<br>• Problem<br>• Test question<br>• Humor<br>• Startling fact<br>• Surveying student knowledge<br>• Relevant to topic<br>• Relevant to audience
**Maintain Attention**	• Make organization clear<br>• Orientation<br>• Guidance<br>• Summarization<br>• Change the stimulus<br>• Video<br>• Demonstration<br>• Role-play
**Promote Learning**	• Limit content to 50% new information<br>• Limit content to 2-3 main points<br>• Select content based on what you want them to remember

Internal Medicine Education Center

The University of Iowa

Interactive Lecturing Action Plan

List 1-2 skills related to interactive lecturing that you would like to improve upon and try when working with learners before the next teaching session.

1.

2.

Art of the Presentation

Internal Medicine Education Center — The University of Iowa

The Art of the Presentation
Checklist

Planning
- ❑ Select topic of interest
- ❑ Identify mentor
- ❑ Confirm logistics: date, location, facility, resources, time allotted for presentation
- ❑ Determine level of learners to gauge depth and breadth of content

Preparing
- ❑ Introduction: tell them what you are going to tell them
  - Capture audience
  - Share main points
- ❑ Body: tell them
  - Explain main points
  - Vary teaching methods
- ❑ Conclusion: tell them what you just told them
  - Summarize main points
  - Allow time for questions

Practicing
- ❑ Be familiar with environment and technical equipment
- ❑ Know material at level deeper than audience
- ❑ Practice slide transition and timing of presentation
- ❑ Deliver talk to mentor and/or colleague for feedback
- ❑ Anticipate questions from audience

Presenting
- ❑ Maintain eye contact
- ❑ Be enthusiastic
- ❑ Acknowledge questions

Internal Medicine Education Center — The University of Iowa

The Art of the Presentation
Timeline

- ❑ Select topic of interest
- ❑ Identify a mentor
- ❑ Establish purpose of talk
- ❑ Consider logistics
- ❑ Know the group’s level of expertise
- ❑ Prepare presentation
- ❑ Practice
- ❑ Deliver talk

Bedside Teaching

Internal Medicine Education Center — The University of Iowa

Bedside Teaching

Teaching in the Patient's Presence

Specific Strategies

BEFORE: prime patient and learners

Prepare the patient/family	• Ask permission<br>• Orient to process
Prime learners	• Set clear, specific goals<br>• Outline roles/involve all<br>• Who leads? Who is backup?<br>• Active observation<br>• Set ground rules and time limits<br>• Anticipate potential problems<br>• Let know when may interrupt
Create safe learning environment	

DURING: teach in patient's presence

- Wash hands
- Introductions
- Teach! Focused, limited
- Role model
- Keep patient-centered
- Reassure patient someone will come back later to answer questions
- Thank the patient/family

Internal Medicine Education Center — The University of Iowa

Bedside Teaching

Teaching in the Patient's Presence

Specific Strategies

AFTER: debrief learners

- Feedback - self-assess
- Answer questions
- Summarize teaching points
- Clarify what needs to be communicated to patient when team member goes back
- More in-depth teaching if desired
- Reflect

Bedside Teaching Action Plan

List 1-2 bedside teaching skills you would like to improve upon and try when working with learners before the next teaching session.

1.

2.

Clinical Reasoning

Internal Medicine Education Center — The University of Iowa

Clinical Reasoning
Priming

Rationale: Priming provides a deliberate means of helping students to think about focused data gathering and differential diagnosis/clinical reasoning even before seeing the patient.

How to do: Initiate a teaching session prior to the patient encounter. “Prime” the student by helping them think about what the initial differential diagnosis is and the key aspects of the history and physical exam

Examples

1. Based on the limited information available, ***outline an initial differential diagnosis.***
2. List the top 5 details you want to elicit from the ***HPI*** to help narrow your differential diagnosis.
3. List 3-5 ***physical findings*** that will be essential to narrowing the differential diagnosis.
4. List 3-5 findings from the history or physical exam that will be essential in determining ***the severity/urgency of the illness***.

Heidenreich C et al. Pediatrics 2000;105(1 pt 3):231-7.

Internal Medicine Education Center — The University of Iowa

Clinical Reasoning
Reverse Presentation

Rationale: By reversing the order of the standard presentation and placing the assessment first, there is an immediate commitment to diagnosis and treatment plan. This allows the preceptor to pay attention to the key features that support or refute the learner’s decision. It also forces the student to select key details to support their assessment.

How to do: Ask learners to present in “ASOAP” format where they briefly give their assessment first and then present the supporting data.

Example

A: This is a 65-yo woman with cough, myalgias and fevers. I think she has influenza. I think we should do a nasal swab for influenza.

S/O: Preceptor listens closely to presentation with the end diagnosis in mind and learner presents information that they think is relevant to the diagnosis.

A/P: Assessment and plan presented in more depth but with description of how the decision was made.

Blankenburg B et al. Pediatric Academic Societies Annual Meeting, May 2011.

Evaluation

Internal Medicine Education Center — The University of Iowa

Evaluating the Learner

G. R. A. D. E.

**G** *(get ready)*	• Set expectations for specific clinical setting<br>• Know the evaluation form
**R** *(review expectations with learner)*	• Review your goals early on<br>• Determine student's skill level<br>• Describe evaluation process
**A** *(assess)*	• Provide feedback<br>• Observe
**D** *(discuss at mid-point of rotation)*	Assess progress in meeting goals
**E** *(evaluation)*	• Fill out CCOM evaluation form in timely manner<br>• Give specific examples of strengths and areas for improvement

University of Virginia Preceptor Development Program. Family Medicine UVa Health System. Module 7

Internal Medicine Education Center — The University of Iowa

Evaluation Action Plan

List 1-2 skills related to evaluation that you would like to improve upon and try when working with learners before the next teaching session.

1.

2.
