# Supplementary material for: Successful implementation of a longitudinal skill-based teaching curriculum for residents
Source: BMC Med Educ. 2021 Jun 15;21:346. doi: 10.1186/s12909-021-02765-x (PMC8207581; doi:10.1186/s12909-021-02765-x)
Supplement: Supplementary file 3 — Additional file 3: Supplemental Table 3. Email distribution of teachings skills information to faculty. [file 12909_2021_2765_MOESM3_ESM.docx]

**Supplemental Table 3.** Email distribution of teachings skills information to faculty.

| 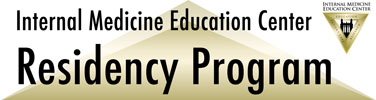 | Announcement |
| --- | --- |
| To: All Faculty and Fellows  At this time we have completed the fourth block of the “X + Y” training schedule. As you may remember from my last note, one component of the Thursday curriculum is the Teaching Skills curriculum which introduces all residents to a new teaching skill every 10 weeks. Residents are provided with pre-session materials and during the teaching session residents are introduced to a particular teaching skill with time for practice. At the end of the session each resident is asked to develop a personal action plan by committing to applying a component of the teaching skill when working with learners. This note is to provide you with the same materials that the residents were introduced to during the second Teaching Skills block.  **Teaching Skills #3: One-Minute Preceptor (see attached pocket card).** We encourage you to review the materials and take the opportunity to role model the skills being taught as well as to ask residents about how they are incorporating these new skills into their teaching.   \| **Teaching Skills #3** \| Topic \| One-Minute Preceptor \| \| --- \| --- \| --- \| \| Pre-Session  Work \| - Review <https://youtube/P0XgABFzcgE>  (if it doesn’t work on a hospital browser try a mobile device) - Think about the steps of the model and reflect on your own practice when you listen to learners’ presentations - Which step of the model is most challenging for you? Is there one that you already feel you do well? \| \| Session Objectives and Handout \| One-Minute Preceptor (see attachment)   - Wrap up the prior “Orienting the Learner” session - Review the steps of the One-Minute Preceptor model and compare it to usual practice - Practice the model in pairs using cases provided - Group discussion of how to apply the model in day-to-day work - Give instructions for deliberate practice of the technique prior to your next teaching Y week \| \| Post-Session Work \| Residents were asked to incorporate deliberate practice into teaching using steps from the One-Minute Preceptor model \|   I will continue to keep you informed regarding the teaching skills residents are learning as we go through the year. I hope this regular email will allow you to reinforce the curriculum and to learn some teaching tips as well!  Manish Suneja  Residency Program Director | |
