# Supplementary material for: Successful implementation of a longitudinal skill-based teaching curriculum for residents
Source: BMC Med Educ. 2021 Jun 15;21:346. doi: 10.1186/s12909-021-02765-x (PMC8207581; doi:10.1186/s12909-021-02765-x)
Supplement: Supplementary file 4 — Additional file 4: Supplemental Table 4. Resident baseline experience and teaching interest. [file 12909_2021_2765_MOESM4_ESM.docx]

**Supplemental Table 4.** Resident baseline experience and teaching interest.

**Teaching Skills Curriculum**

Name (please print): ____________________________ Date:______________________

1. Indicate your current level of training: ⃝ R1 ⃝ R2 ⃝ R3 ⃝ R4 ⃝ R5
2. Current training program: ⃝ Categorical ⃝ Preliminary
3. Indicate settings where you have had previous teaching experience (check all that apply):
   1. ⃝ Bedside teaching
   2. ⃝ Large group teaching/lecturing
   3. ⃝ Simulation
   4. ⃝ Small group teaching
   5. ⃝ Other:_______________________
4. Indicate all level(s) of learner you have taught:
   1. ⃝ Medical student
   2. ⃝ Resident
   3. ⃝ Fellow
   4. ⃝ Staff
   5. ⃝ Other:______________________
5. I took part in a formal teaching course during medical school:    ⃝ Yes     ⃝ No

If yes, what medical school did you attend:________________________________

1. Do you see teaching as part of your future career?

| Will not be part of my career  1 | 2 | 3 | 4 | Will be part of my career  5 |
| --- | --- | --- | --- | --- |

1. Rate your interest in teaching.

| Not at all interested  1 | 2 | 3 | 4 | Extremely interested  5 |
| --- | --- | --- | --- | --- |
