# Supplementary material for: Successful implementation of a longitudinal skill-based teaching curriculum for residents
Source: BMC Med Educ. 2021 Jun 15;21:346. doi: 10.1186/s12909-021-02765-x (PMC8207581; doi:10.1186/s12909-021-02765-x)
Supplement: Supplementary file 5 — Additional file 5: Supplemental Table 5. Teaching skills confidence survey. [file 12909_2021_2765_MOESM5_ESM.docx]

**Supplemental Table 5.** Teaching skills confidence survey.

**Teaching Skills Curriculum**

Name: ____________________________________ Date: ______________________

Directions: For the teaching skills/characteristics listed below, circle the value that indicates how confident you feel in performing each of them.

|  | **Not at all confident** |  |  |  | **Very confident** |
| --- | --- | --- | --- | --- | --- |
| Facilitate a small group | 1 | 2 | 3 | 4 | 5 |
| Identify important skills for teachers | 1 | 2 | 3 | 4 | 5 |
| Evaluate learners | 1 | 2 | 3 | 4 | 5 |
| Identify key steps in teaching a procedure | 1 | 2 | 3 | 4 | 5 |
| Use wait time when questioning learners | 1 | 2 | 3 | 4 | 5 |
| Give feedback consistently | 1 | 2 | 3 | 4 | 5 |
| Ask learners to identify their learning goals | 1 | 2 | 3 | 4 | 5 |
| Choose appropriate ways to assess learners | 1 | 2 | 3 | 4 | 5 |
| Deal with challenging learners | 1 | 2 | 3 | 4 | 5 |
| Create a positive learning environment | 1 | 2 | 3 | 4 | 5 |
| Show respect for the learner | 1 | 2 | 3 | 4 | 5 |
| Choose appropriate methods for delivering content | 1 | 2 | 3 | 4 | 5 |
| Orient a new learner | 1 | 2 | 3 | 4 | 5 |
| Clearly convey your expectations | 1 | 2 | 3 | 4 | 5 |
