# Supplementary material for: Successful implementation of a longitudinal skill-based teaching curriculum for residents
Source: BMC Med Educ. 2021 Jun 15;21:346. doi: 10.1186/s12909-021-02765-x (PMC8207581; doi:10.1186/s12909-021-02765-x)
Supplement: Supplementary file 6 — Additional file 6: Supplemental Table 6. Teaching skills self-assessment. [file 12909_2021_2765_MOESM6_ESM.docx]

## Supplemental Table 6. Teaching skills self-assessment.

## TEACHING SKILLS POST-PROGRAM QUESTIONNAIRE

## Name:________________________________ Date:________________________

Using the following scale, answer the following statements in regard to how closely they describe your instructional experience and behavior at the beginning of the Resident Teaching Skills curriculum and now.

1 = I do not do this

2 = generally not able to do this

3 = somewhat able to do this

4 = quite competent in doing this

5 = highly competent in doing this

During my educational interactions with learners:

I convey my expectations with regard to learning, performance and behavior.

| Before the Teaching Skills curriculum | Now |
| --- | --- |
| 1 2 3 4 5 | 1 2 3 4 5 |

I discuss with the learner her/his goals during the rotation.

| Before the Teaching Skills curriculum | Now |
| --- | --- |
| 1 2 3 4 5 | 1 2 3 4 5 |

I demonstrate my interest in teaching and allot time for it.

| Before the Teaching Skills curriculum | Now |
| --- | --- |
| 1 2 3 4 5 | 1 2 3 4 5 |

I create a positive and supportive learning environment.

| Before the Teaching Skills curriculum | Now |
| --- | --- |
| 1 2 3 4 5 | 1 2 3 4 5 |

I show support and respect for learners.

| Before the Teaching Skills curriculum | Now |
| --- | --- |
| 1 2 3 4 5 | 1 2 3 4 5 |

I choose appropriate methods for learning the material.

| Before the Teaching Skills curriculum | Now |
| --- | --- |
| 1 2 3 4 5 | 1 2 3 4 5 |

I ask questions that encourage learners to think about the medical issue.

| Before the Teaching Skills curriculum | Now |
| --- | --- |
| 1 2 3 4 5 | 1 2 3 4 5 |

I give frequent and constructive feedback.

| Before the Teaching Skills curriculum | Now |
| --- | --- |
| 1 2 3 4 5 | 1 2 3 4 5 |

I provide an opportunity for learners to observe and participate in clinically relevant procedures.

| Before the Teaching Skills curriculum | Now |
| --- | --- |
| 1 2 3 4 5 | 1 2 3 4 5 |

I engage learners in discussions about medical issues.

| Before the Teaching Skills curriculum | Now |
| --- | --- |
| 1 2 3 4 5 | 1 2 3 4 5 |

I clearly communicate information about the student’s performance during the rotation.

| Before the Teaching Skills curriculum | Now |
| --- | --- |
| 1 2 3 4 5 | 1 2 3 4 5 |

I feel comfortable stating “I’m not sure” when I don’t know the answer.

| Before the Teaching Skills curriculum | Now |
| --- | --- |
| 1 2 3 4 5 | 1 2 3 4 5 |

I coach learners through new procedures instead of doing them myself.

| Before the Teaching Skills curriculum | Now |
| --- | --- |
| 1 2 3 4 5 | 1 2 3 4 5 |

I actively listen to the student when they are presenting information.

| Before the Teaching Skills curriculum | Now |
| --- | --- |
| 1 2 3 4 5 | 1 2 3 4 5 |

I ask students for feedback on my teaching skills and abilities.

| Before the Teaching Skills curriculum | Now |
| --- | --- |
| 1 2 3 4 5 | 1 2 3 4 5 |
