# Supplementary material for: Successful implementation of a longitudinal skill-based teaching curriculum for residents
Source: BMC Med Educ. 2021 Jun 15;21:346. doi: 10.1186/s12909-021-02765-x (PMC8207581; doi:10.1186/s12909-021-02765-x)
Supplement: Supplementary file 7 — Additional file 7: Supplemental Table 7. Teaching skills curriculum evaluation. [file 12909_2021_2765_MOESM7_ESM.docx]

**Supplemental Table 7.** Teaching skills curriculum evaluation.

| **Statement** | **Strongly disagree (1)** | **2** | **3** | **4** | **Strongly agree**  **(5)** |
| --- | --- | --- | --- | --- | --- |
| The amount of work I was expected to complete was reasonable |  |  |  |  |  |
|  |  |  |  |  |  |
| I found the practice/role-play during the sessions to be helpful in learning specific teaching skills being emphasized |  |  |  |  |  |
|  |  |  |  |  |  |
| The pocket cards summarized the teaching skills being highlighted |  |  |  |  |  |
|  |  |  |  |  |  |
| I refer to the cards in my teaching |  |  |  |  |  |
|  |  |  |  |  |  |
| The topics chosen were helpful in my development as a teacher |  |  |  |  |  |
|  |  |  |  |  |  |
| The sessions provided me with a longitudinal comprehensive teaching skills curriculum |  |  |  |  |  |
|  |  |  |  |  |  |
| The “Y” week teaching skills curriculum provides a comprehensive program for my development as a teacher |  |  |  |  |  |
|  |  |  |  |  |  |
| I have seen the teaching skills role modeled by faculty |  |  |  |  |  |
|  |  |  |  |  |  |
| Faculty have provided feedback on the teaching skills I was introduced to during the Resident Teaching Skill curriculum |  |  |  |  |  |
|  |  |  |  |  |  |
