# Supplementary material for: Successful implementation of a longitudinal skill-based teaching curriculum for residents
Source: BMC Med Educ. 2021 Jun 15;21:346. doi: 10.1186/s12909-021-02765-x (PMC8207581; doi:10.1186/s12909-021-02765-x)
Supplement: Supplementary file 8 — Additional file 8: Supplemental Table 8A. Confidence score after first year of participation in curriculum by program year. Supplemental Table 8B. Confidence score after second year of participation in curriculum by program year. [file 12909_2021_2765_MOESM8_ESM.docx]

**Supplemental Table 8A.** Confidence score after first year of participation in curriculum by program year.

| **Question** | **Program year** | **n** | **1** | | **2** | | **3** | | **4** | | **5** | | **p-value*** |
| --- | --- | --- | --- | --- | --- | --- | --- | --- | --- | --- | --- | --- | --- |
|  |  |  | **Count** | **%** | **Count** | **%** | **Count** | **%** | **Count** | **%** | **Count** | **%** |  |
| Ask learners to identify their learning goals | 1 | 56 | 0 | *0* | 5 | *8.9* | 12 | *21.4* | 21 | *37.5* | 18 | *32.1* |  |
|  | 2 | 20 | 0 | *0* | 0 | *0* | 3 | *15.0* | 10 | *50.0* | 7 | *35.0* | 0.57 |
|  | 3 | 19 | 0 | *0* | 0 | *0* | 3 | *15.8* | 12 | *63.2* | 4 | *21.1* |  |
| Choose appropriate methods for delivering content | 1 | 56 | 0 | *0* | 2 | *3.6* | 6 | *10.7* | 40 | *71.4* | 8 | *14.3* |  |
|  | 2 | 20 | 0 | *0* | 0 | *0* | 3 | *15.0* | 10 | *50.0* | 7 | *35.0* | 0.13 |
|  | 3 | 19 | 0 | *0* | 0 | *0* | 1 | *5.3* | 14 | *73.7* | 4 | *21.1* |  |
| Choose appropriate ways to assess learners | 1 | 56 | 1 | *1.8* | 2 | *3.6* | 20 | *35.7* | 29 | *51.8* | 4 | *7.1* |  |
|  | 2 | 20 | 0 | *0* | 0 | *0* | 5 | *25.0* | 13 | *65.0* | 2 | *10.0* | 0.07 |
|  | 3 | 19 | 0 | *0* | 0 | *0* | 4 | *21.1* | 13 | *68.4* | 2 | *10.5* |  |
| Clearly convey your expectations | 1 | 56 | 0 | *0* | 0 | *0* | 16 | *28.6* | 24 | *42.9* | 16 | *28.6* |  |
|  | 2 | 20 | 0 | *0* | 0 | *0* | 4 | *20.0* | 6 | *30.0* | 10 | *50.0* | 0.10 |
|  | 3 | 19 | 0 | *0* | 0 | *0* | 1 | *5.3* | 12 | *63.2* | 6 | *31.6* |  |
| Create a positive learning environment | 1 | 56 | 0 | *0* | 0 | *0* | 5 | *8.9* | 25 | *44.6* | 26 | *46.4* |  |
|  | 2 | 20 | 0 | *0* | 0 | *0* | 3 | *15* | 6 | *30* | 11 | *55* | 0.57 |
|  | 3 | 19 | 0 | *0* | 0 | *0* | 1 | *5.3* | 8 | *42.1* | 10 | *52.6* |  |
| Deal with challenging learners | 1 | 56 | 1 | *1.8* | 11 | *19.6* | 31 | *55.4* | 11 | *19.6* | 2 | *3.6* |  |
|  | 2 | 20 | 0 | *0* | 3 | *15.0* | 8 | *40.0* | 9 | *45.0* | 0 | *0* | 0.01 |
|  | 3 | 19 | 0 | *0* | 1 | *5.3* | 8 | *42.1* | 9 | *47.4* | 1 | *5.3* |  |
| Evaluate learners | 1 | 56 | 0 | *0* | 0 | *0* | 12 | *21.4* | 36 | *64.3* | 8 | *14.3* |  |
|  | 2 | 20 | 0 | *0* | 0 | *0* | 1 | *5.0* | 16 | *80.0* | 3 | *15.0* | 0.88 |
|  | 3 | 19 | 0 | *0* | 0 | *0* | 6 | *31.6* | 11 | *57.9* | 2 | *10.5* |  |
| Facilitate a small group | 1 | 56 | 1 | *1.8* | 3 | *5.4* | 19 | *33.9* | 28 | *50.0* | 5 | *8.9* |  |
|  | 2 | 20 | 0 | *0* | 0 | *0* | 3 | *15.0* | 11 | *55.0* | 6 | *30.0* | 0.01 |
|  | 3 | 19 | 0 | *0* | 0 | *0* | 3 | *15.8* | 14 | *73.7* | 2 | *10.5* |  |
| Give feedback consistently | 1 | 56 | 0 | *0* | 3 | *5.4* | 20 | *35.7* | 24 | *42.9* | 9 | *16.1* |  |
|  | 2 | 20 | 0 | *0* | 1 | *5.0* | 3 | *15.0* | 13 | *65.0* | 3 | *15.0* | 0.03 |
|  | 3 | 19 | 0 | *0* | 0 | *0* | 3 | *15.8* | 11 | *57.9* | 5 | *26.3* |  |
| Identify important skills for teachers | 1 | 56 | 1 | *1.8* | 0 | *0* | 18 | *32.1* | 29 | *51.8* | 8 | *14.3* |  |
|  | 2 | 20 | 0 | *0* | 0 | *0* | 5 | *25.0* | 12 | *60.0* | 3 | *15.0* | 0.05 |
|  | 3 | 18 | 0 | *0* | 0 | *0* | 2 | *11.1* | 11 | *61.1* | 5 | *27.8* |  |
| Orient a new learner | 1 | 56 | 0 | *0* | 1 | *1.8* | 6 | *10.7* | 36 | *64.3* | 13 | *23.2* |  |
|  | 2 | 20 | 0 | *0* | 0 | *0* | 4 | *20.0* | 10 | *50.0* | 6 | *30.0* | 0.52 |
|  | 3 | 19 | 0 | *0* | 0 | *0* | 2 | *10.5* | 11 | *57.9* | 6 | *31.6* |  |
| Show respect for the learner | 1 | 56 | 0 | *0* | 0 | *0* | 3 | *5.4* | 16 | *28.6* | 37 | *66.1* |  |
|  | 2 | 20 | 0 | *0* | 0 | *0* | 2 | *10.0* | 6 | *30.0* | 12 | *60.0* | 0.74 |
|  | 3 | 19 | 0 | *0* | 0 | *0* | 0 | *0* | 5 | *26.3* | 14 | *73.7* |  |
| Use wait time when questioning learners | 1 | 56 | 1 | *1.8* | 2 | *3.6* | 11 | *19.6* | 33 | *58.9* | 9 | *16.1* |  |
|  | 2 | 20 | 0 | *0* | 1 | *5.0* | 3 | *15.0* | 14 | *70.0* | 2 | *10.0* | 0.48 |
|  | 3 | 19 | 0 | *0* | 1 | *5.3* | 1 | *5.3* | 14 | *73.7* | 3 | *15.8* |  |

*p-value from Jonckheere-Terpstra test to test for ordered differences in confidence score among program years

**Supplemental Table 8B.** Confidence score after second year of participation in curriculum by program year.

| **Question** | **Program year** | **n** | **1** | | **2** | | **3** | | **4** | | **5** | | **p-value*** |
| --- | --- | --- | --- | --- | --- | --- | --- | --- | --- | --- | --- | --- | --- |
|  |  |  | **Count** | **%** | **Count** | **%** | **Count** | **%** | **Count** | **%** | **Count** | **%** |  |
| Ask learners to identify their learning goals | 2 | 31 | 1 | *3.2* | 1 | *3.2* | 7 | *22.6* | 7 | *22.6* | 15 | *48.4* | 0.62 |
|  | 3 | 17 | 0 | *0.0* | 0 | *0.0* | 2 | *11.8* | 7 | *41.2* | 8 | *47.1* |  |
| Choose appropriate methods for delivering content | 2 | 31 | 0 | *0.0* | 0 | *0.0* | 3 | *9.7* | 23 | *74.2* | 5 | *16.1* | 0.47 |
|  | 3 | 17 | 0 | *0.0* | 0 | *0.0* | 3 | *17.6* | 8 | *47.1* | 6 | *35.3* |  |
| Choose appropriate ways to assess learners | 2 | 31 | 0 | *0.0* | 2 | *6.5* | 4 | *12.9* | 23 | *74.2* | 2 | *6.5* | 0.17 |
|  | 3 | 17 | 0 | *0.0* | 0 | *0.0* | 3 | *17.6* | 9 | *52.9* | 5 | *29.4* |  |
| Clearly convey your expectations | 2 | 31 | 0 | *0.0* | 1 | *3.2* | 4 | *12.9* | 16 | *51.6* | 10 | *32.3* | 0.09 |
|  | 3 | 17 | 0 | *0.0* | 0 | *0.0* | 0 | *0.0* | 8 | *47.1* | 9 | *52.9* |  |
| Create a positive learning environment | 2 | 31 | 0 | *0.0* | 0 | *0.0* | 1 | *3.2* | 11 | *35.5* | 19 | *61.3* | 0.59 |
|  | 3 | 17 | 0 | *0.0* | 0 | *0.0* | 1 | *5.9* | 7 | *41.2* | 9 | *52.9* |  |
| Deal with challenging learners | 2 | 31 | 1 | *3.2* | 2 | *6.5* | 12 | *38.7* | 15 | *48.4* | 1 | *3.2* | 0.88 |
|  | 3 | 17 | 0 | *0.0* | 1 | *5.9* | 8 | *47.1* | 8 | *47.1* | 0 | *0.0* |  |
| Evaluate learners | 2 | 31 | 1 | *3.2* | 0 | *0.0* | 5 | *16.1* | 20 | *64.5* | 5 | *16.1* | 0.07 |
|  | 3 | 17 | 0 | *0.0* | 0 | *0.0* | 3 | *17.6* | 6 | *35.3* | 8 | *47.1* |  |
| Facilitate a small group | 2 | 31 | 1 | *3.2* | 0 | *0.0* | 5 | *16.1* | 22 | *71.0* | 3 | *9.7* | 0.01 |
|  | 3 | 17 | 0 | *0.0* | 0 | *0.0* | 1 | *5.9* | 9 | *52.9* | 7 | *41.2* |  |
| Give feedback consistently | 2 | 31 | 0 | *0.0* | 1 | *3.2* | 10 | *32.3* | 20 | *64.5* | 0 | *0.0* | 0.09 |
|  | 3 | 17 | 0 | *0.0* | 0 | *0.0* | 5 | *29.4* | 7 | *41.2* | 5 | *29.4* |  |
| Identify important skills for teachers | 2 | 31 | 1 | *3.2* | 0 | *0.0* | 1 | *3.2* | 23 | *74.2* | 6 | *19.4* | 0.63 |
|  | 3 | 17 | 0 | *0.0* | 0 | *0.0* | 2 | *11.8* | 10 | *58.8* | 5 | *29.4* |  |
| Orient a new learner | 2 | 31 | 0 | *0.0* | 1 | *3.2* | 4 | *12.9* | 14 | *45.2* | 12 | *38.7* | 0.23 |
|  | 3 | 17 | 0 | *0.0* | 0 | *0.0* | 2 | *11.8* | 5 | *29.4* | 10 | *58.8* |  |
| Show respect for the learner | 2 | 31 | 0 | *0.0* | 0 | *0.0* | 0 | *0.0* | 5 | *16.1* | 26 | *83.9* | 0.12 |
|  | 3 | 17 | 0 | *0.0* | 0 | *0.0* | 1 | *5.9* | 5 | *29.4* | 11 | *64.7* |  |
| Use wait time when questioning learners | 2 | 31 | 0 | *0.0* | 0 | *0.0* | 3 | *9.7* | 19 | *61.3* | 9 | *29.0* | 0.65 |
|  | 3 | 17 | 0 | *0.0* | 0 | *0.0* | 1 | *5.9* | 10 | *58.8* | 6 | *35.3* |  |

*p-value from Wilcoxon rank-sum exact test
