# Supplementary material for: Successful implementation of a longitudinal skill-based teaching curriculum for residents
Source: BMC Med Educ. 2021 Jun 15;21:346. doi: 10.1186/s12909-021-02765-x (PMC8207581; doi:10.1186/s12909-021-02765-x)
Supplement: Supplementary file 9 — Additional file 9: Supplemental Table 9. AAMC graduation questionnaire: “Residents provide effective teaching during clerkship.” [file 12909_2021_2765_MOESM9_ESM.docx]

**Supplemental Table 9.** AAMC graduation questionnaire: “Residents provide effective teaching during clerkship.”

| **Internal Medicine** | **Year** | **Strongly disagree (1)** | **Disagree (2)** | **Neutral (3)** | **Agree (4)** | **Strongly agree (5)** | **Count** |
| --- | --- | --- | --- | --- | --- | --- | --- |
| All medical schools | 2018 | 1.0 | 1.9 | 4.3 | 24.7 | 68.2 | 15,321 |
| Iowa-Carver | 2018 | 0.0 | 0.0 | 2.4 | 20.8 | 76.8 | 125 |
| All medical schools | 2016 | 0.9 | 1.6 | 4.2 | 25.4 | 67.9 | 14,159 |
| Iowa-Carver | 2016 | 0.0 | 1.6 | 4.9 | 29.3 | 64.2 | 123 |
